# Utilization of whole health services among veterans with spinal cord injuries and disorders (SCI/D): Early insights from the VA SCI/D system of care

**DOI:** 10.1080/10790268.2023.2183325

**Published:** 2023-05-30

**Authors:** Erik S. Wallen, Jennifer L. Sippel, Meaghan E. Park, Bella Etingen, Frances M. Weaver, Timothy P. Hogan, Bridget M. Smith, Barbara G. Bokhour, Itala M. Wickremasinghe

**Affiliations:** 1 Spinal Cord Injuries and Disorders National Program Office, Veterans Health Administration, Washington, DC, USA; 2 Center of Innovation for Complex Chronic Healthcare (CINCCH), Edward Hines Jr. VA Hospital, Hines, Illinois, USA; 3 Public Health Sciences, Parkinson School of Health Sciences and Public Health, Loyola University, Maywood, Illinois, USA; 4 Center for Healthcare Organization and Implementation Research (CHOIR), VA Bedford Healthcare System, Bedford, Massachusetts, USA; 5 Department of Population and Data Sciences, UT Southwestern Medical Center, Dallas, Texas, USA; 6 Northwestern University Feinberg School of Medicine, Chicago, Illinois, USA; 7 Department of Population and Quantitative Health Sciences, University of Massachusetts Medical School, Worcester, Massachusetts, USA

**Keywords:** . Patient-centered care, Spinal cord injuries and disorders, Whole Health, Veterans, Veterans health administration

## Abstract

**Context/Objective:**

Our objective was to describe early participation in Whole Health programs across the Veterans Health Administration (VHA) Spinal Cord Injuries and Disorders (SCI/D) System of Care.

**Design:**

Retrospective analysis of VHA administrative data.

**Setting:**

The VHA SCI/D System of Care.

**Participants:**

Veterans with SCI/D included in the FY2019 cumulative VHA SCI/D Registry cohort with living status during FY2017, FY2018, and FY2019.

**Interventions:**

N/A.

**Outcome Measures:**

We assessed the number of encounters and unique Veterans with SCI/D, and the percent of Veterans with SCI/D, who utilized each Whole Health (WH) program available in VA.

**Results:**

Utilization of WH Pathway and well-being Programs increased from 62 encounters to 1703 encounters between FY2017 and FY2019 (representing 0.09% to 3.13% of Veterans with SCI/D). Utilization of chiropractic care rose from 130 encounters to 418 encounters during the same time period. Similarly, utilization of complementary and integrative health programs increased from 886 encounters to 2655 encounters (representing 1.09% to 3.11% of Veterans; FY2017 to 2019). We also report utilization of specific WH programs.

**Conclusion:**

Participation in WH services has been increasing among Veterans with SCI/D who receive health care from the VHA SCI/D System of Care. However, utilization among Veterans with SCI/D remains low overall, and targeted efforts to increase WH program reach are needed. Additional information about the relative effectiveness of different strategies to support WH implementation is also needed, to ensure strategies likely to have the most impact are prioritized.

## Introduction

The Department of Veterans Affairs, Veterans Health Administration (VHA), the largest integrated healthcare system in the United States, is currently engaged in a system-wide transformation of its healthcare delivery to adopt the Whole Health approach to care.

Whole Health is only the latest in a long history of patient-centered approaches in medical rehabilitation. During and after World War II, Ludwig Guttman championed a patient-centered approach that focused on just on preserving life but also on giving persons with SCI a purpose in life (1). Mark Ozer, a pioneer in patient-centered rehabilitation focused on problems and outcomes related to the needs of each patient (2).

The transformation to Whole Health is a significant change in the philosophy and practice of healthcare (3, 4). The practice of Whole Health is centered around providing holistic, comprehensive healthcare to patients that addresses their values, goals, and preferences, as well as their health conditions. Rather than focus solely on a patient's diagnoses, this shift in healthcare culture and practice emphasizes what matters most to the patient(3). Through the incorporation of strategies such as Personalized Health Planning, health coaching, and complementary and integrative health (CIH) services (*e.g.* yoga, massage, acupuncture), Whole Health care delivery is intended to be patient-centered – that is, personalized to the needs, preferences and unique life circumstances of each individual patient (5). The VHA Whole Health approach to care can be conceptualized by three over-arching themes: empowering, equipping, and treating Veterans. Veterans are empowered through the Whole Health Pathway to explore their Mission, Aspiration, and Purpose and to develop a Personal Health Plan. Well-being programs equip Veterans with new skills that support self-care, including CIH approaches, self-care practices, and Whole Health coaching. Whole Health Clinical Care treats Veterans in VHA facilities, the community, or both, by clinicians trained in Whole Health. Importantly, the provision of care that is patient-centered may be associated with improvements in healthcare quality (6).

Veterans with complex chronic conditions, (*e.g.* those with spinal cord injuries or disorders (SCI/D)), may particularly benefit from the Whole Health model of care (*e.g.* use of CIH Services) (7–10). Following injury, persons with SCI/D experience physical (*e.g.* chronic pain, pressure ulcers, spasticity, bowel and bladder complications) and psychosocial (*e.g.* depression, anxiety, posttraumatic stress disorder) difficulties at high rates (11-18), and often experience unique life circumstances that impact mobility, access and healthcare needs and influence both physical and psychosocial well-being.

### Implementation of Whole Health within the VHA SCI/D system of care

VHA provides lifelong care for Veterans with SCI/D, which is overseen by the SCI/D National Program Office. The VHA SCI/D System of Care serves over 18,000 Veterans with SCI/D through a Hub and Spokes system, with 25 SCI/D Centers (Hubs), that provide comprehensive SCI/D care, and over 115 Spokes sites that provide primary care and some specialty care. Whole Health implementation has been a priority for the VHA SCI/D System of Care since 2018. Recognizing that successful transformation of a health care system requires change at all levels of the organization (19), the SCI/D National Program Office began rolling Whole Health out to VHA SCI/D System of Care using a staged approach.

To kick off its Whole Health implementation efforts, the SCI/D Program Office hosted a conference in FY 2018 for key SCI/D Center leadership. During this meeting, representatives from the VHA Office of Patient Centered Care and Cultural Transformation (OPCC&CT), the lead program office responsible for Whole Health implementation across the VHA system, presented education about Whole Health, related programs, and the applicability of Whole Health to SCI/D. Additionally, attendees from each SCI/D Center collaborated to develop SCI/D Center-specific action plans that addressed facility priorities, baseline activities, intra-facility collaborative opportunities, actions planned, timelines, and metrics.

Following the conference, VHA and SCI/D national leadership have provided support to the VHA SCI/D System of Care for Whole Health implementation, including resources available through the VHA intranet, virtual meetings, and other national networking opportunities. SCI/D clinicians and leaders throughout the VHA SCI/D System of Care have shared information about opportunities and successes with Whole Health implementation for Veterans with SCI/D. Communication and cross-site collaboration have been facilitated by the creation and maintenance of a national SCI/D Whole Health email group. In addition, a comprehensive SCI/D Whole Health coding and tracking guide has been developed, which helps monitor Whole Health implementation progress across the VHA SCI/D System of Care and utilization of Whole Health programs among Veterans with SCI/D. These tracking efforts include workload reports of Whole Health activities that are customizable and that allow the viewing of specific information about Whole Health program use in particular regions. Please see Table 1 for a summary of Whole Health programs available in VHA.

**Table 1:** VHA Whole Health Programs.

Classification	Definition	Available Programs
The Pathway	Group learning opportunities and support services that introduce Veterans to the Whole Health concept and available services, support them in identifying what matters most to them, and help them set health-related goals in line with these identified values. Pathway services can be led by Peers or care team members.	Introduction to Whole Health Taking Charge of My Life and Health Whole Health Partner (Individual)
Well-being Programs	Programs that are offered across the VHA system of care that aim to empower Veterans to support their health and well-being.	Advance Care Planning (Group) Empower Veteran Program (EVP) – Acceptance and Commitment Therapy EVP – Mindful Movement EVP – Whole Health Whole Health Coaching (Individual or Group) Whole Health Education
List 1 Complementary and Integrative Health (CIH) Approaches	CIH that must be available (either within VHA facilities or through community-based referrals), as appropriate, to Veterans who are interested in participating.	Acupuncture, Battlefield Acupuncture (BFA), Battlefield Auricular Acupressure (BAA) Biofeedback Clinical Hypnosis Guided Imagery Massage Therapy Meditation/ Mantram Repetition/Mindfulness Based Stress Reduction (MBSR)/Mindfulness Other than MBSR Tai Chi / Qi Gong Yoga
Classification	Definition	Programs Encompassed
List 2 CIH Approaches	CIH programs that VHA facilities can offer; availability is not mandatory.	Native American Healing Pilates Animal-Assisted Therapy Reiki Relaxation Techniques Therapeutic or Healing Touch
Other Well-Being	Approved by VA but not on list 2	Expressive Arts Movement Therapy
Chiropractic Care	Services provided by a Doctor of Chiropractic Medicine, examples include therapeutic exercises, join manipulation and mobilization, and soft tissue therapies.	Chiropractic Care
Whole Health Clinical Care	Clinicians partner with Veterans to equip them to enhance their health and well-being in alignment with shared goals and the Veteran's mission, aspiration, or purpose, integrating Pathway, Well-being, and CIH as appropriate.	Whole Health Clinical Care

Notably, specific Whole Health programs implemented at any given VHA site are determined at a regional and/or local level based on resource availability, staff and contractor skill sets, and an assessment of what services would be most appropriate for the Veteran population of that site.

With their comprehensive array of SCI/D-specific services, SCI/D Centers (Hubs) have resources that enable them to provide Whole Health programs within the SCI/D service. SCI/D System of Care Spoke facilities similarly have had the opportunity to establish Whole Health programs. Veterans served at both SCI/D Hubs and Spokes may also access clinically appropriate Whole Health services offered outside of the SCI/D service line (*i.e.* those that also are available to the general Veteran population at that site).

Some literature suggests that efforts to increase facets of patient-centered care (*e.g.* shared decision-making, empathic patient/provider interactions) may be beneficial for Veterans with SCI/D (20, 21). However, little information exists to-date about how to support the adoption of Whole Health services among individuals with SCI/D. The overall objective of this paper is to examine trends in early participation of Whole Health programs among Veterans with SCI/D who receive VHA SCI/D care.

## Materials and methods

### Design

We conducted a retrospective database analysis of utilization of Whole Health services among Veterans with SCI/D.

### Participants/Setting

Veterans encompassed in our analyses include individuals with SCI/D who received specialty inpatient, outpatient, home care, and/or telehealth services in the VHA SCI/D System of Care during fiscal years (FY) 2017, 2018, and/or 2019, and were included in the VHA SCI/D Registry (22).

### Data sources

Veterans with SCI/D were identified through VHA's SCI/D Registry (VSR) (22). Data on Veteran utilization of Whole Health services were obtained from the VHA administrative healthcare workload data in the VHA Corporate Data Warehouse. In FY2018, SCI/D Centers provided multiple updates detailing where Veterans with SCI/D were receiving Whole Health services (within an SCI/D Center or through another department at the VHA Medical Center) and which Whole Health services Veterans were receiving. Some Centers also gathered site-specific information, to enhance local implementation efforts. In order to assure data standardization during the Whole Health implementation process, national implementation leaders from the SCI/D National Program Office subsequently facilitated a comprehensive coding and tracking guide for SCI/D Whole Health services workload capture for Veterans with SCI/D receiving care at each of the 25 SCI/D Centers grounded in guidance and standards developed by OPCC&CT. Specific clinics and CHAR4 codes (which further defines clinic workload by program, service, specialty, team or provider), some of which are SCI/D or Whole Health specific, are used to identify use of clinics and utilization of specific health care services. A stop code is a VHA term that characterizes VHA clinics. Specifically, they indicate the work group responsible for providing the specific set of clinic products and serve as a stable identification method that can be used to compare costs between facilities.

### Analyses

We examined the number of encounters, the number of Veterans with SCI/D, and the percent of Veterans with SCI/D (living or who passed away in a FY) who received: (1) at least one Whole Health service in a Whole Health affiliated clinic (Table 2), and (2) utilization of individual Whole Health programs in a clinic assigned one of the Whole Health affiliated CHAR4 codes (Tables 3–5). We examined these data in three FYs: FY2017 (pre-implementation), FY2018 (initial implementation), and FY2019 (continued implementation). This work was reviewed by a VHA Institutional Review Board and determined to be quality improvement, exempting it from further oversight.

**Table 2:** Utilization of Whole Health Among Veterans with SCI/D (Stop Code Workload Data).

Whole Health Service	Utilization: FY 2017 (*n* = 15,639)			Utilization: FY 2018 (*n* = 16,168)			Utilization: FY 2019 (*n* = 17,520)		
	Veterans	% of Veterans	Encounters	Veterans	% of Veterans	Encounters	Veterans	% of Veterans	Encounters
Whole Health Pathway and Well-being Programs	14	0.09%	62	215	1.33%	672	548	3.13%	1,703
Chiropractic Care	34	0.22%	130	35	0.22%	163	92	0.53%	418
Complementary & Integrative Health	171	1.09%	886	258	1.60%	1,104	545	3.11%	2,655

**Table 3:** Utilization of Whole Health Pathway and Well-being Programs Among Veterans with SCI/D (CHAR4 Workload Data).

	Utilization: FY 2017 (*n* = 15,639)			Utilization: FY 2018 (*n* = 16,168)			Utilization: FY 2019 (*n* = 17,520)		
**Whole Health Pathway Programs**	Veterans	% of Veterans	Encounters	Veterans	% of Veterans	Encounters	Veterans	% of Veterans	Encounters
Introduction to Whole Health	0	0.00%	0	1	0.01%	1	66	0.38%	68
Taking Charge of My Life and Health	2	0.01%	13	8	0.05%	28	52	0.30%	113
Whole Health Partner (Individual)	3	0.02%	4	3	0.02%	3	12	0.07%	16
**Whole Health Well-being Programs**	Veterans	% of Veterans	Encounters	Veterans	% of Veterans	Encounters	Veterans	% of Veterans	Encounters
Advance Care Planning (Group)	0	0.00%	0	0	0.00%	0	0	0.0%	0
Empower Veterans Program (EVP) – Acceptance and Commitment Therapy	0	0.00%	0	6	0.04%	36	2	0.01%	6
EVP – Mindful Movement	0	0.00%	0	6	0.04%	42	4	0.02%	33
EVP – Whole Health	0	0.00%	0	5	0.03%	27	1	0.01%	1
Whole Health Coaching (Group)	4	0.03%	22	10	0.06%	31	28	0.16%	105
Whole Health Coaching (Individual)	7	0.04%	11	45	0.28%	105	79	0.45%	211
Whole Health Education	105	0.67%	389	133	0.82%	527	172	0.98%	614

**Table 4:** Utilization of List 1 and List 2 CIH Approaches Among Veterans with SCI/D (CHAR4 Workload Data).

	FY 2017 (*n* = 15,639)			FY 2018 (*n* = 16,168)			FY 2019 (*n* = 17,520)		
List 1 CIH Approaches	Veterans	% of Veterans	Encounters	Veterans	% of Veterans	Encounters	Veterans	% of Veterans	Encounters
Acupuncture	129	0.82%	636	196	1.21%	640	278	1.59%	1,182
Battlefield Acupuncture	22	0.14%	38	31	0.19%	79	38	0.22%	114
Biofeedback	1	0.01%	1	13	0.08%	32	8	0.05%	24
Clinical Hypnosis	1	0.01%	1	1	0.01%	2	2	0.01%	4
Guided Imagery	0	0.00%	0	1	0.01%	4	1	0.01%	6
Massage Therapy	3	0.02%	30	3	0.02%	100	54	0.31%	333
Meditation	1	0.01%	1	1	0.01%	5	2	0.01%	5
Qi Gong	1	0.01%	10	2	0.01%	11	2	0.01%	5
Tai Chi	8	0.05%	98	8	0.05%	33	35	0.20%	162
Yoga	15	0.10%	37	37	0.23%	179	54	0.31%	340
Mantram Repetition	3	0.02%	22	0	0.00%	0	0	0.00%	0
Mindfulness Based Stress Reduction (MBSR)	7	0.04%	21	6	0.04%	21	6	0.03%	15
Mindfulness Other than MBSR	7	0.04%	9	116	0.72%	169	253	1.44%	326
**List 2 CIH Approaches**	Veterans	% of Veterans	Encounters	Veterans	% ofVeterans	Encounters	Veterans	% ofVeterans	Encounters
Animal-Assisted Therapy	0	0.00%	0	1	0.01%	1	19	0.11%	43
Pilates	2	0.01%	8	2	0.00%	9	0	0.00%	0
Reiki	11	0.07%	102	9	0.06%	63	9	0.05%	58
Relaxation Techniques	1	0.01%	3	4	0.02%	11	12	0.07%	32
Therapeutic or Healing Touch	3	0.02%	5	4	0.02%	10	11	0.06%	37
**Other Well-Being**	Veterans	% of Veterans	Encounters	Veterans	% of Veterans	Encounters	Veterans	% of Veterans	Encounters
Expressive Arts	6	0.04%	28	6	0.04%	16	32	0.18%	116
Movement Therapy	47	0.30%	299	68	0.42%	483	226	1.29%	1,557

**Table 5:** Utilization of Chiropractic Care and Whole Health Clinical Care Among Veterans with SCI/D (CHAR4 Workload Data).

** **	**FY 2017 (*n* = 15,639)**			**FY 2018 (*n* = 16,168)**			**FY 2019 (*n* = 17,520)**		
	Veterans	% of Veterans	Encounters	Veterans	% of Veterans	Encounters	Veterans	% of Veterans	Encounters
Chiropractic Care	29	0.19%	115	28	0.17%	136	67	0.38%	282
Whole Health Clinical Care	714	4.57%	2,614	812	5.02%	2,975	953	5.44%	3,800

## Results

Below we present national-level utilization rates of Whole Health programs among Veterans with SCI/D.

### Overall utilization (Table 2)

Overall, use of Whole Health Services among Veterans with SCI/D steadily increased over the three FYs, with the lowest rates seen in the pre-implementation period (FY2017) and rising in the initial (FY2018) and continued (FY2019) implementation periods. Specifically, utilization of Whole Health Pathway and well-being Programs increased from 62 encounters representing 0.09% of Veterans with SCI/D (FY2017) to 672 encounters (1.33%, FY2018) to 1,703 encounters (3.13%, FY2019). Utilization of chiropractic care rose from 130 and 163 encounters representing 0.22% Veterans (in FY2017 and 2018, respectively) to 418 encounters (0.53% Veterans with SCI/D, FY2019). Similarly, utilization of CIH programs increased from 886 encounters representing 1.09% Veterans with SCI/D (FY2017) to 1,104 encounters (1.60%) in FY2018 and 2655 encounters (3.11%) in, FY2019.

### Whole Health pathway and Whole Health well-being programs (Table 3)

**Figure 1:**
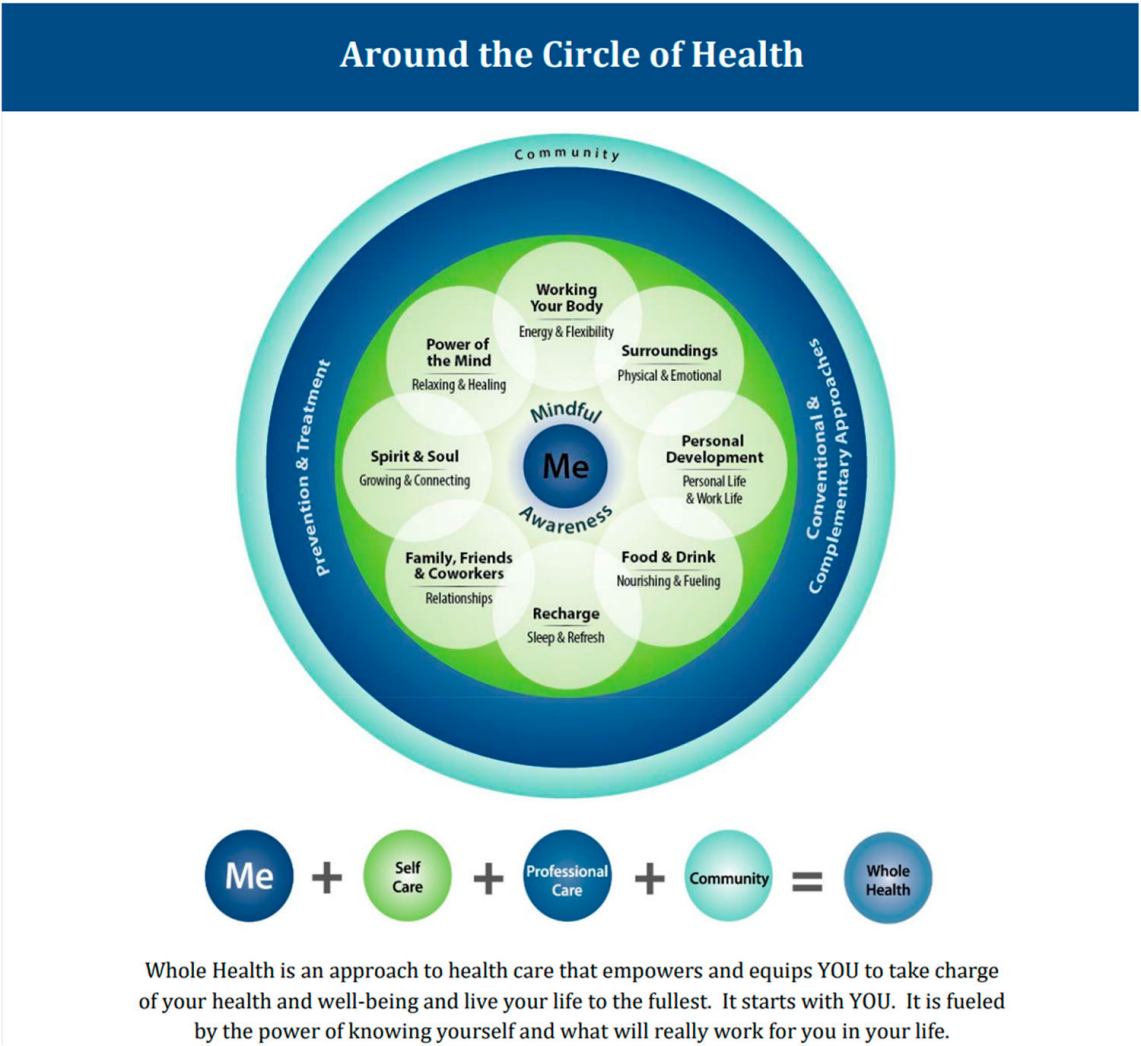
Circle of Health from VHA's Whole Health Skill Building Protocol.

Use of Whole Health Pathway programs among Veterans with SCI/D was very low in FY2017 and has recently begun to increase. Specifically, while there was no use of the Introduction to Whole Health program among Veterans with SCI/D in FY2017, utilization increased to 68 encounters representing 0.38% of Veterans with SCI/D in FY2019. Introduction to Whole Health is a 2-hour that exposes participants to the foundational concepts of Whole Health, allows time for self-care and self-exploration, and for completion of a Personal Health Inventory. Facilitators are often Veterans trained conduct this class. Similarly, there were 113 Taking Charge of My Life and Health (TCMLH) program encounters (representing 0.30% of Veterans with SCI/D) in FY2019 and 16 Whole Health Partner program encounters (representing 0.07% of Veterans with SCI/D) in FY2019. TCMLH is a longer-term Whole Health group program where Veterans can participate in self-exploration in areas in their lives they wish to enhance, learn about well-being services, and create a personalized health plan with SMART goals and action steps that will help them attain these goals. The most widely utilized Whole Health Well-being programs were Whole Health Coaching, which is offered in group and individual settings, and Whole Health Education. Whole Health Education is defined as any group or individual educational offering aligned with whole health and focused around one of the eight components of proactive health and well-being (Figure 1). Whole Health Education can be offered in individual or group formats; they are more often provided in a group setting because (1) the benefits are expanded when Veterans have the opportunity to help each other and draw on each other's experiences and encouragement; and (2) it is more cost-effective.

Use of Group Whole Health Coaching increased from 22 encounters, representing 0.03% of Veterans with SCI/D in FY2017, to 31 encounters (0.06% of Veterans) in FY2018 to 105 encounters (0.16% of Veterans with SCI/D, FY2019). Similarly, use of Individual Whole Health Coaching increased from 11 encounters representing 0.04% of Veterans with SCI/D (FY2017) to 105 encounters (0.28% of Veterans with SCI/D, FY2018) to 211 encounters (0.45% Veterans with SCI/D, FY2019). Use of the Whole Health Education program increased from 389 encounters representing in FY2017 (0.67% of Veterans with SCI/D) to 527 encounters by0.82% of Veterans with SCI/D in FY2018 to 614 encounters in FY2019 (0.98% of Veterans).

### List 1 and list 2 CIH approaches (Table 4)

List 1 CIH approaches are those, subjective to clinical caveats, and given the level of evidence, must be made available to Veterans across VHA, either within a VA medical facility or in the community. The most widely utilized List 1 CIH programs were acupuncture, massage therapy and yoga. Use of acupuncture increased from 636 encounters in FY2017 to 640 encounters in FY2018 and 1,182 encounters in FY2019 (0.82%, 1.21% and 1.59%, of Veterans with SCI/D, respectively). Utilization of massage therapy increased from 30 to 100 to 333 encounters from FY2017 to 2019, representing 0.02%, 0.02% and 0.31% of Veterans with SCI/D, respectively. Similarly, participation in yoga increased across time, increasing from 37 encounters to 179 encounters and 340 encounters (representing 0.10%, 0.23% and 0.31% of Veterans). List 2 CIH approaches are those that are optional to provide in VHA and are generally considered safe. Included in this analysis under List 2 are CIH approaches that were approved by VA and delivered by other services such as Recreational Therapy and modalities, such as mantram repetition, which are not specified on List 1. The most widely utilized List 2 CIH programs were movement therapy, mindfulness other than mindfulness-based stress reduction (MBSR), and expressive arts. Use of movement therapy increased from 299 encounters in FY2017 to 483 in FY2018 and 1,557 encounters in FY2019 (representing 0.30% 0.42%, and 1.29% of Veterans with SCI/D). Similarly, utilization of mindfulness other than MBSR increased from 9 to 169 to 326 encounters (representing 0.04%, 0.72% and 1.44% of Veterans with SCI/D from FY2017 to FY2019). Use of expressive arts increased from 28 and 16 encounters in FY2017 and FY2018, to 116 encounters in FY2019 (0.04% to 0.18% of Veterans with SCI/D).

### Chiropractic care and Whole Health clinical care (Table 5)

Use of chiropractic care services increased from 115 encounters in FY2017 to 136 encounters in, FY2018 and 282 encounters in FY2019 (0.38% of Veterans with SCI/D). Documented Whole Health clinical care increased across the 3 FYs, increasing from 4.57% of Veterans with SCI/D (2614 encounters in FY2017) to 5.02% of Veterans (2,975 encounters) in FY2018 and 5.44% of Veterans in FY2019.

## Discussion

This manuscript is among the first to describe the utilization of Whole Health programs among individuals with SCI/D who receive care in a large integrated healthcare system. Encouragingly, utilization data reveal a clear pattern of increasing program use over time. While the current project cannot definitively address the reasons that program utilization is increasing, we suspect that potential contributing factors could include expanded availability of services, increased Veteran and/or clinician interest, bolstered commitment to the Whole Health culture change at one or multiple levels of the VA organization, and/or the broader societal acceptance and utilization of Whole Health programs (19). These data indicate that while there has been some uptake in Whole Health among Veterans with SCI/D, which is promising, utilization is still quite low. Although this increasing utilization trend reflects overall trends in Whole Health use across the 18 VHA facilities that piloted Whole Health across the nation (20), it suggests that additional implementation efforts across the SCI/D System of Care are warranted, particularly those supporting further program adoption; such efforts could be supported by examinations of what barriers may exist to Whole Health participation and what strategies may facilitate greater uptake of Whole Health by Veterans with SCI/D.

Several strategies were used to support Whole Health implementation within the VHA SCI/D System of Care. For instance, the use of clinical champions (individuals who support an implementation effort from within the implementation setting) can effectively facilitate successful implementation (23). Likewise, facilitating environments where collaborative learning and work can take place is an effective strategy by which healthcare institutions can enact practice change (24-26). Importantly, difficulties with documentation of program use has been noted as a key barrier to successful implementation of Whole Health programs (21). As such, the VHA SCI/D system of care instituted a comprehensive Whole Health workload tracking program as part of this implementation effort is highly important to assess implementation success and future monitoring of related program utilization. Finally, the present evaluation represents a partnership between VHA health services researchers and two VHA program offices: The Office of Patient Centered Care & Cultural Transformation (OPCC&CT) and the Spinal Cord Injuries & Disorders (SCI/D) National Program Office. Partnered projects involving both researchers and policy-makers (in VHA, these include national program offices) can support overcoming implementation barriers and streamlining system-level change (in this case Whole Health expansion) (27).

While increases in Whole Health program utilization over time among Veterans with SCI/D have been notable, the overall utilization of Whole Health Programs within this population is low and varies widely across Whole Health services. During our observed time-frame, we found much lower use of CIH programs than did Taylor *et al.* (26). Our data cannot delineate the reasons behind the observed utilization rates; however, a number of factors may have impacted program use within our population. For instance, these early rates of utilization of Whole Health programs may have been a product of the relatively new nature of this initiative, with significant implementation efforts beginning in FY 2018; it may have taken facilities some time to roll out various programs, and more time still to inform clinicians and Veterans of those programs. Further, there may have been variation across VHA facilities in the availability of Whole Health programs, resources available for them, and/or the settings (*e.g.* primary care, mental health) offering Whole Health services. This variation may have increased or decreased availability of programs for Veterans with SCI/D outside of those offered by the SCI/D Centers. Issues related to access may negatively impact reach of Whole Health programs; (28) such issues may be particularly salient for Veterans with SCI/D, who often face unique physical and access challenges. Finally, some Whole Health programs may be more or less appropriate to incorporate into a Veteran's treatment plans, and Veteran and provider preferences may impact program utilization as well.

Literature suggests that Veterans find the use of CIH to be helpful (27) and early findings from a large-scale evaluation of Whole Health implementation found a wide range of benefits to patients with chronic pain (20). Accordingly, efforts should be taken to ensure that Whole Health programs are accessible for individuals with disabilities. One strategy for accomplishing this important goal is adapting Whole Health programs for use by this vulnerable patient cohort. Adaptation for specialized implementation settings and populations is scientifically sound and an appropriate and impactful strategy for optimizing program implementation and reach (29) and may offer an important means for VHA to improve reach of Whole Health to Veterans with SCI/D and other disabilities. An example of a specialized implementation setting for Veterans with SCI/D is virtual care. Some Whole Health programs have been piloted through these methods, increasing access and reach; expansion of these early trials is recommended moving forward. Moreover, additional work is needed to assess the factors driving Whole Health utilization and any existing disparities in access to Whole Health services that may be experienced by this population.

### Limitations

This program was being rolled out across VHA and this takes time to implement in SCI/D Centers. The structure of the tracking mechanisms that were used are limited by the available Whole Health and CIH clinic codes and CHAR4 workload codes, creating the potential for coding errors. The data available for this analysis was limited, and does not address potential changes in utilization that may have resulted from the COVID-19 pandemic. Further, our data does not provide insight about the reasons behind observed utilization, including the influence of temporal trends.

## Conclusions

The VHA SCI/D System of Care is enthusiastic and devoted to the implementation of Whole Health for Veterans with SCI/D and has used several established strategies to support the implementation of Whole Health across the VHA SCI/D System of Care, including clinical champions, learning collaboratives, and strong national and local-level leadership support. Whole Health implementation efforts within the VHA SCI/D System of Care appear to be yielding an increase in use of Whole Health programs among Veterans with SCI/D. While the absolute utilization of these programs remains low, each encounter represents system-level change towards both the practical aspects of integrating Whole Health services into VHA SCI/D care and a culture change within the VHA SCI/D System of Care and VHA at large to support the Whole Health model of care delivery. These changes represent shifting perceptions among both care team members and Veterans about how to support Veteran health care and well-being. However, our utilization data also indicate that targeted efforts are needed to increase Whole Health program reach for Veterans with SCI/D. This speaks to the importance of studying the relative effectiveness of the different implementation strategies that the VHA SCI/D System of Care is currently leveraging, so that those likely to have the most impact are prioritized and supported across the system. Additional efforts could include targeted Whole Health program adaptation to ensure appropriateness of program content for Veterans with disabilities.
